# Torque–Cadence Profile and Maximal Dynamic Force in Cyclists: A Novel Approach

**DOI:** 10.3390/s24061997

**Published:** 2024-03-21

**Authors:** Víctor Rodríguez-Rielves, David Barranco-Gil, Ángel Buendía-Romero, Alejandro Hernández-Belmonte, Enrique Higueras-Liébana, Jon Iriberri, Iván R. Sánchez-Redondo, José Ramón Lillo-Beviá, Alejandro Martínez-Cava, Raúl de Pablos, Pedro L. Valenzuela, Jesús G. Pallarés, Lidia B. Alejo

**Affiliations:** 1 Human Performance and Sports Science Laboratory, Faculty of Sport Sciences, University of Murcia, 30720 Murcia, Spain; victor@entrenamiento.pro (V.R.-R.); angel.buendiaromero@uclm.es (Á.B.-R.); alejandro.hernandez7@um.es (A.H.-B.); enriquehiglie@gmail.com (E.H.-L.); jr.lillo@ua.es (J.R.L.-B.); alejandro.martinez12@um.es (A.M.-C.); 2 Faculty of Sport Sciences, European University of Madrid, 28670 Madrid, Spain; david.barranco@universidadeuropea.es (D.B.-G.); ivanrodriguez.trainer@gmail.com (I.R.S.-R.); raulbq98@gmail.com (R.d.P.); lidia.brea@universidadeuropea.es (L.B.A.); 3 GENUD Toledo Research Group, Faculty of Sports Sciences, University of Castilla-La Mancha, 45071 Toledo, Spain; 4 Jumbo Visma Professional Cycling Team, 5215 MV Den Bosch, The Netherlands; j-iriberri@euskadi.eus; 5 Physical Activity and Health Research Group (PaHerg), Research Institute of Hospital 12 de Octubre (Imas12), 28041 Madrid, Spain; pedro.valenzuela92@gmail.com; 6 Department of Systems Biology, University of Alcalá, 28871 Madrid, Spain

**Keywords:** assessment, cycling, force, testing, laboratory

## Abstract

We aimed to determine the feasibility, test–retest reliability and long-term stability of a novel method for assessing the force (torque)-velocity (cadence) profile and maximal dynamic force (MDF) during leg-pedaling using a friction-loaded isoinertial cycle ergometer and a high-precision power-meter device. Fifty-two trained male cyclists completed a progressive loading test up to the one-repetition maximum (1RM) on a cycle ergometer. The MDF was defined as the force attained at the cycle performed with the 1RM-load. To examine the test–retest reliability and long-term stability of torque–cadence values, the progressive test was repeated after 72 h and also after 10 weeks of aerobic and strength training. The participants’ MDF averaged 13.4 ± 1.3 N·kg^−1^, which was attained with an average pedal cadence of 21 ± 3 rpm. Participants’ highest power output value was attained with a cadence of 110 ± 16 rpm (52 ± 5% MDF). The relationship between the MDF and cadence proved to be very strong (R^2^ = 0.978) and independent of the cyclists’ MDF (*p* = 0.66). Cadence values derived from this relationship revealed a very high test–retest repeatability (mean SEM = 4 rpm, 3.3%) and long-term stability (SEM = 3 rpm, 2.3%); despite increases in the MDF following the 10-week period. Our findings support the validity, reliability and long-term stability of this method for the assessment of the torque–cadence profile and MDF in cyclists.

## 1. Introduction

Force–velocity evaluations, usually performed by incremental loading tests, enable a comprehensive evaluation of muscle mechanical capabilities [1]. For resistance (e.g., in kg), an athlete has to overcome increases, and so does the force he/she has to apply to move it. However, the difference between this force applied by the athlete and that represented by the resistance becomes smaller and smaller along the incremental test, which decreases the resulting velocity (whether linear or radial). Thus, a relationship by plotting the main two variables (i.e., force and velocity) or others derived (e.g., power) can be modeled [2,3]. Although these tests are typically used in the context of strength exercises (e.g., knee extension, squat, bench press), they can also be applied in other muscle tasks such as leg pedaling [4]. To this effect, several methods have been used for evaluating the force (torque)-velocity (cadence) profile in bicycle ergometer exercises, including the completion of a series of isokinetic efforts [5] or a single isoinertial effort after a stationary start [6,7]. These approaches, however, have some limitations, including low specificity or a small number of resulting data (in the case of isokinetic and single-sprint tests, respectively) [4].

Several studies have assessed the torque–cadence profile during bicycling through short-duration maximal efforts performed against different resistive forces [4,8,9,10,11]. These methods show a high test–retest reliability for estimating the theoretical maximal values of torque (T_0_ or F_0_), pedal cadence and power output, as well as the (‘optimal’) cadence associated with the maximal power output produced [10,11]. Of note, these indicators have been positively associated with cycling performance [8] and the torque–cadence profile can be used to identify imbalances in the lower-limb mechanical capacities, thereby potentially allowing training programs to be prescribed based on each individual’s needs (e.g., high-load or high-cadence training targeting improvements in the maximal levels of torque or cadence, respectively) [12]. Despite the practical applicability of the torque–cadence profile, its long-term stability in relative terms (i.e., the cadence associated with the percentage of the individual maximal torque value) is unknown. If present, long-term stability, which has been already studied for other upper- and lower-limb isoinertial exercises [13,14], can reinforce the applicability of the torque–cadence profile as an evaluation and training prescription method in the sport of cycling.

Another indicator commonly determined during the assessment of the force–velocity profile in muscle strength exercises is the maximal dynamic force (MDF, usually represented by the one-repetition maximum, 1RM) [15]. However, to the best of our knowledge, the MDF has not yet been assessed during pedaling, even if this parameter might provide potentially useful information. Indeed, the determination of MDF enables the force applied in each pedaling stroke to be expressed during training or racing (i.e., as a percentage of the MDF). For instance, during training sessions aimed at improving torque production capacity, a training modality that has gained popularity in recent years [16,17,18], researchers and coaches could also prescribe and monitor training loads based on the percentages of MDF in addition to other ‘classical’ indicators such as relative intensity based on heart rate or power output ‘zones’ that do not accurately identify the actual medium-to-high (>50% MDF) intensity efforts [19].

Considering all the above, the present study aimed to determine the feasibility, test–retest reliability, and long-term stability of a novel methodological procedure for determining the torque–cadence profile and the MDF during leg pedaling using a high-precision power-meter sensor mounted on a friction-loaded isoinertial cycle ergometer in trained cyclists.

## 2. Materials and Methods

### 2.1. Participants

Fifty-two male cyclists volunteered to participate in the study (age [mean ± standard deviation (SD)] 29.3 ± 8.3 years; training experience, 17.5 ± 7.3 years; height, 174 ± 5 cm; body mass, 71.9 ± 6.9 kg). As reflected by the results of previous testing in our laboratory [19] (maximum oxygen uptake = 63.8 ± 6.7 mL·kg^−1^·min^−1^, peak power output = 5.4 ± 0.7 W∙kg^−1^), cyclists were considered highly trained [20]. All subjects were instructed to maintain their normal diet during the study period, as well as to refrain from performing high-intensity exercise or ingesting caffeine or other stimulants 48 h before each testing session. They were informed of the study procedures and provided written informed consent. The study was approved by the Ethical Committee of the Local University (ID: 4135/2022), and all procedures were conducted following the standards established by the Declaration of Helsinki and its later amendments.

### 2.2. Experimental Approach

All the participants performed three familiarization sessions with the testing procedures. To study whether the torque–cadence profile was dependent on individual MDF levels, the participants were ranked according to their MDF and divided into three tertile groups as follows: low (*n* = 17), medium (*n* = 18) and high MDF (*n* = 18), respectively. On the other hand, seven subjects from each group (i.e., total = 21 participants) were randomly selected to analyze the test–retest reliability (*n* = 10) and long-term stability (*n* = 11), as described below.

### 2.3. Procedures

**Figure 1:**
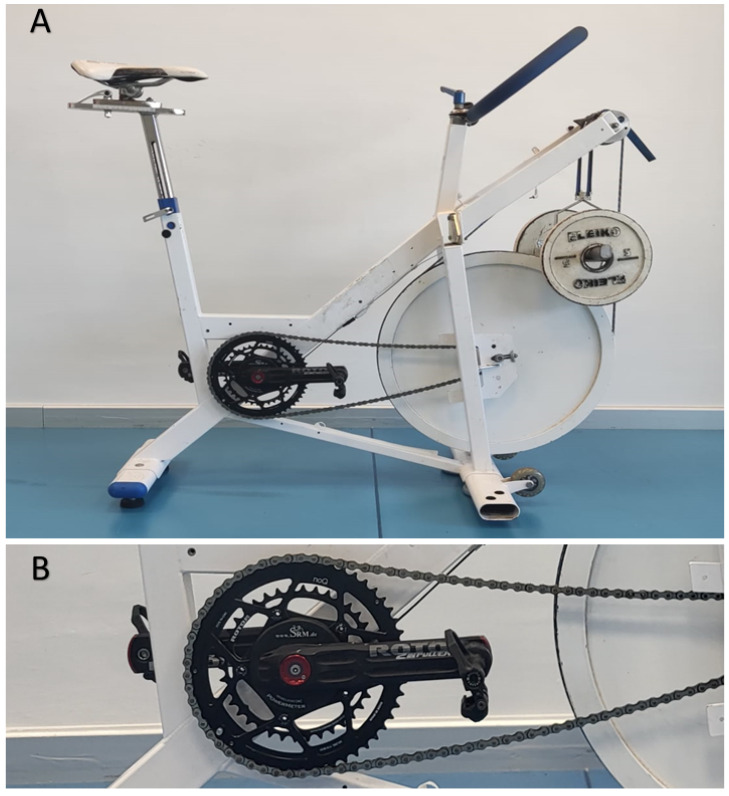
Cycle-ergometer (**A**) and power-meter used in the study (**B**).

**Figure 2:**
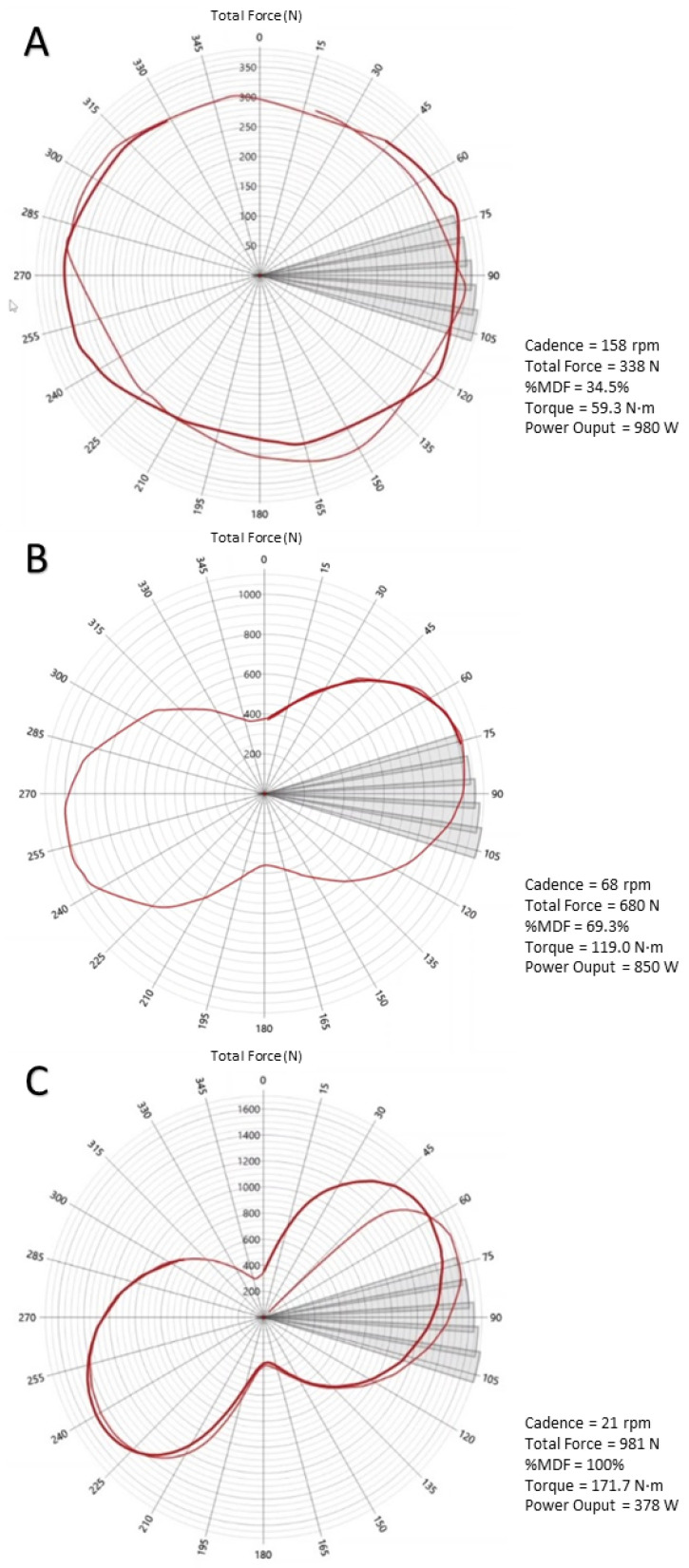
Screenshots of the power-meter software analysis with examples of the applied total force attained by a study participant at each crank angle (50 Hz) for a pedaling cycle against a low (~35% of maximal dynamic force [MDF]) (**A**), moderate (~70% of MDF) (**B**) or maximum resistive force (100 of MDF, i.e., one-repetition maximum) (**C**).

*Feasibility.* All incremental pedaling tests were conducted in a friction-loaded isoinertial cycle ergometer (Monark^©^ 874E; Varberg, Sweden) equipped with a 175 mm crank. The position of the saddle (height and setback) and handlebar (reach and drop) was individually adjusted to replicate the participant’s own bike (Figure 1A). The test started with the crank of the preferred leg at 45° relative to the vertical position. The initial load (2 kp) was progressively increased by 0.5 to 3 kp in each trial through the addition of calibrated disks (Eleiko, Sport AB; Halmstad, Sweden) until the heaviest load was reached above which the cyclist could no longer properly perform a whole (360°) pedaling cycle (i.e., 1RM; the precision of 0.5 kp). Load increments were individualized so that participants reached 1RM in less than 8 attempts, interspersed by 5-min rests (i.e., 2-min, free-cadence active recovery against 1 kp followed by 3-min passive recovery). Participants were required to perform a 5-s all-out effort with each load. Only the pedal cycle (i.e., a complete cycle with both legs) associated with the highest cadence was used for the subsequent analyses. In addition, the force (N), torque (N·m^−1^, considering the crack length), and power output (W) achieved during the highest cadence cycle were registered. The MDF was defined as the force attained with the 1RM-load. In order to directly measure the pedaling force and crank position during each pedaling cycle and trial, a recently validated high-precision power meter (Rotor 2INpower, Madrid, Spain; 50 Hz) [21] was adapted to the bottom bracket of the cycle ergometer (Figure 1B). The power meter was calibrated at the beginning of each testing session following the manufacturer’s instructions. A specific software (Rotor INPower Software 2.2) was used for the analysis of force, torque, cadence and power output data (Figure 2).

*Test–retest reliability.* The above-described incremental test (using the same absolute loads in kp) was repeated after 72 h (test–retest reliability).

*Long-term stability*. The above-described incremental test (same absolute loads) was also repeated after a 10-week combined endurance and resistance training program. In addition to their habitual cycling endurance training (10.4 ± 0.8 h per week), participants underwent a standardized resistance training intervention 3 days per week (5 sets of 7 free-weight squat repetitions at 70% of 1RM per session) during the aforementioned program. The cyclist’s 1RM in the full squat exercise was accurately estimated by the lifting velocity as detailed elsewhere [14]. During the training program, both relative intensity (70% 1RM) and intra-set volume (half of the possible repetitions per set) were programmed using the level of effort strategy, which has been proven to be a precise, reliable, and practical alternative to velocity-based training [22].

### 2.4. Statistical Analyses

Standard statistical methods were used for the calculation of the mean, standard deviation (SD), coefficient of determination (R^2^), standard error of the estimate (SEE), and 95% confidence interval (CI). Relationships between variables were studied by fitting second-order polynomials to the data. The standard error of measurement (SEM) was calculated in absolute and relative ([100 × SEM]/mean) terms from the square root of the mean square error in a repeated-measures ANOVA test. The normality of the data was verified using the Shapiro–Wilk test. Cross-sectional differences between MDF-tertile groups were examined through a one-way ANOVA test with Scheffé’s post hoc comparisons. Differences between the test–retest results (test–retest reliability) and pre- and post-training results (long-term stability) were analyzed with paired *t*-tests. The level of significance was set at 0.05. Analyses were performed using SPSS software version 20.0 (IBM Corporation; Armonk, NY, USA).

*Test–retest reliability.* The above-described incremental test (using the same absolute loads in kp) was repeated after 72 h (test–retest reliability).

*Long-term stability*. The above-described incremental test (with the same absolute loads) was also repeated after a 10-week combined endurance and resistance training program. In addition to their habitual cycling endurance training (10.4 ± 0.8 h per week), participants underwent a standardized resistance training intervention 3 days per week (5 sets of 7 free-weight squat repetitions at 70% of 1RM per session) during the aforementioned.

## 3. Results

### 3.1. Feasibility

**Figure 3:**
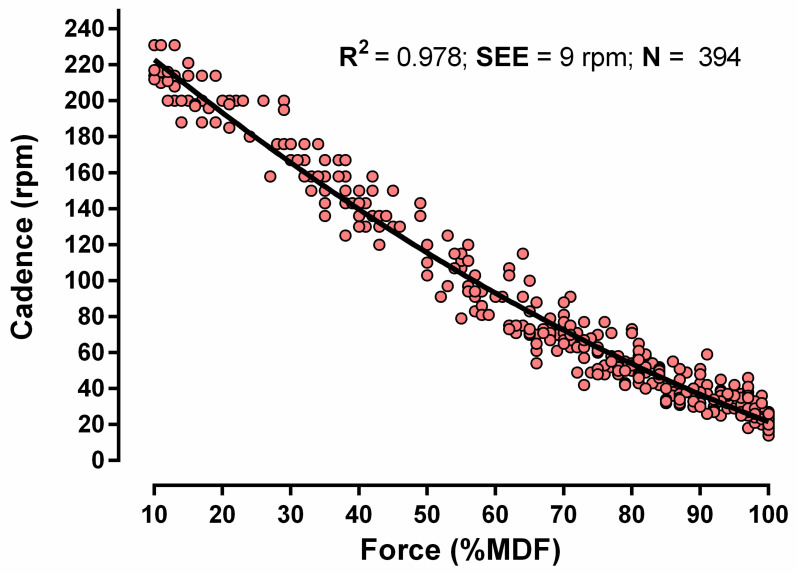
Force (expressed relative to the maximum dynamic force [MDF])–cadence relationship (*n* = 52 participants).

On average, participants performed 7 ± 1 attempts until reaching their MDF, which was successfully determined in all of them. The loads used during the incremental pedaling test ranged between 2.0 and 21.5 kp. No adverse events were noted during the tests. After plotting pedaling cadence, on the one hand, against the % of MDF and, on the other, fitting a second-order polynomial to all data points, a very close relationship between these two variables was found (R^2^ = 0.978; SEE = 9 rpm; Figure 3). Individual curve fits for each test yielded an R^2^ value of 0.980 ± 0.013 (95% confidence interval, 0.976 to 0.983). A prediction equation to estimate the relative torque (% of MDF) from cadence (rpm) could be obtained (R^2^ = 0.975; SEE = 4.5% of MDF) as follows:

the % of MDF = (0.0007595 × rpm^2^) − (0.6163 × rpm) + 111.4

**Figure 4:**
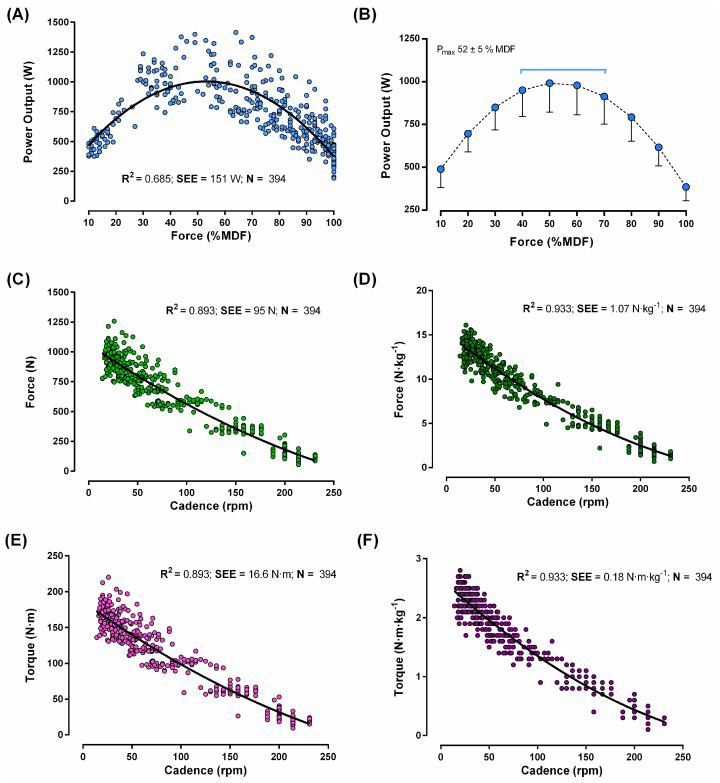
Power output to force (expressed relative to the maximum dynamic force [MDF]) relationship (individual and average data shown in (**A**,**B**)), force–cadence relationship (**C**,**D**), and torque–cadence relationship (**E**,**F**) (*n* = 52 participants).

The torque–power output (panels A and B), force–cadence (panels C and D) and torque–cadence (panels E and F) relationships are shown in Figure 4. Participants’ MDF (961 ± 108 N or 13.4 ± 1.3 N·kg^−^^1^) was achieved with a load of 17 ± 2 kp and a cadence of 21 ± 3 rpm. Participants attained the highest power output with a cadence of 110 ± 16 rpm, corresponding to 52 ± 5% of their MDF. The polynomial equations showed a good fit (R^2^ ≥ 0.893) for the force–cadence and torque–cadence relationships.

Finally, no significant differences were observed for the average (including the whole force–cadence spectrum, *p* = 0.528, F-value = 0.301) or minimum cadence (i.e., that attained at the MDF, *p* = 0.487, F-value = 0.287) across participants with different MDF levels (Table 1).

### 3.2. Test–Retest Reliability

The MDF (969 ± 74 N vs. 965 ± 65 N, *p* > 0.05) and its associated cadence (23 ± 4 rpm vs. 22 ± 2 rpm, *p* > 0.05) were similar on days 1 and 2. When analyzing the cadence attained at different percentages of MDF, the results showed a very high test-rest repeatability (mean SEM = 4 rpm, 3.3%) (Table 2).

### 3.3. Long-Term Stability

Although the MDF of the participants who underwent the 10-week resistance program significantly increased with training (966 ± 76 N vs. 1001 ± 92 N at pre- and post-training, respectively, *p* = 0.013), values from the %MDF–cadence relationship remained stable from pre- to post-training (SEM = 4 rpm, 2.3%) (Table 2).

## 4. Discussion

In the present study, we propose a novel test for the assessment of the torque–cadence profile and MDF during pedaling. Through the proposed equation, the relative resistive load (expressed as a % of MDF) produced during a given effort could be estimated by attending to the attained cadence. Our findings support an overall high test–retest reliability of the force–velocity profile, as well as high stability in the face of performance changes or different levels of cyclists’ MDF. Thus, this test might be useful for prescribing or identifying relative intensities and for monitoring training-induced changes in different zones of the force–velocity curve.

Previous studies have implemented methodological procedures for the assessment of the force–cadence profile during cycling. Rudsits et al. [4] determined this profile through six sprints against increasing external loads, eliciting torque values from 0 to 4 N·m·kg^−1^, which resulted in cadences ranging between ~41 and ~214 rpm. Of note, the authors concluded that a robust assessment of the torque–velocity profile during pedaling required recording a large number of pedal cycles completed over a wide range of cadences [4]. However, these authors mostly focused on how the testing and modeling procedures can influence the torque–cadence profile and did not assess the test–retest reliability or long-term stability as we did here.

García-Ramos et al. [10] also determined the force–velocity profile through 5–6 sprints against increasing external resistive forces between 0.4 N·kg^−1^ (172 rpm) and 1.3 N·kg^−1^ (83 rpm). Interestingly, the authors observed a higher test–retest reliability for the cadence associated with the lightest compared to the heaviest loads, respectively, which was confirmed in the present study. For this reason, these authors recommended using two distant but relatively light loads (corresponding to >110 rpm) when applying the so-called ‘two-point method’ for the estimation of the force–velocity profile [10]. In a subsequent study, the same research group confirmed that the two-point method, using 180–200 rpm and 110–125 rpm, could be a reliable procedure for assessing the force–velocity profile during pedaling [11].

In the present study, we assessed the torque–velocity profile using the widest range of cadences assessed to date (from ~22 to ~220 rpm). In this regard, although in line with García-Ramos et al., we found lower reliability with the heavier loads (e.g., SEM > 5% with cadences < 90 rpm), and the overall force–velocity profile appeared highly reliable (SEM of 2 to 3%). Moreover, we observed a very high consistency in this profile, with a given cadence representing a similar relative load despite between- and within-subject variations for the MDF (as shown in Table 1 and Table 2, respectively). These results might support the validity of the torque–cadence profile for identifying potential limitations or weaknesses in an individual’s profile and for the assessment of training-induced changes. For instance, García-Ramos et al. [12] found that 6 weeks of both heavy- and light-load sprint training induced a shift in the slope of the torque–cadence profile. However, those individuals who trained with heavy loads improved their maximum torque levels to a greater extent than those who trained using a light load, whereas the opposite trend was observed for the highest cadences.

In the present study, we also propose a novel indicator, such as the MDF during pedaling, defined as the maximum force that can be produced during a whole pedaling cycle. This parameter seems to correspond, at least in the present cohort, to a cadence of 21–22 rpm regardless of individuals’ MDF levels, as confirmed in both within- and between-subject analyses. Thus, our findings might support the validity of the percentage of the MDF as an indicator of the relative load of efforts as performed during training or competition, as well as during specific strength training stimuli (e.g., the so-called ‘torque’ training) [16,17,18]. This could be of particular relevance for research purposes, allowing to match relative training loads during cycling using the velocity of muscle contractions (i.e., pedal cadence), similar to what is typically performed in resistance exercises such as leg squat, bench press, or prone bench pulls (i.e., velocity-based training) [14,23]. This practical application for cycling can be exemplified by recent studies assessing the effects of the so-called ‘torque’ training (i.e., performing short-duration bouts at low cadences [40–60 rpm] to increase torque production capacity) [16,17,18]. However, the authors of these studies [16,17,18] could not quantify the relative loads of these bouts with respect to the participants’ MDF (Figure 2), and therefore, whether these training sessions actually elicited high individual torque levels remains unknown.

It is worth emphasizing that we propose a relatively simple and economical procedure for the assessment of the force–velocity profile during cycling, as well as for the determination of a novel parameter such as the MDF. Our results showed that the % of the MDF–cadence relationship is reliable and stable over time, regardless of the changes or different levels of cyclists’ MDF. In practice, the very close adjustment we found for this relationship would allow cyclists: (i) to determine the percentage of the MDF that is being used during every training or competition effort and (ii) to program the target cadence to train at a planned percentage of the MDF. Moreover, the cadence achieved against the same load (in kp) pre-and post-training could be measured (iii) for practically quantifying changes in cyclist’s performance—e.g., the pre-post training differences of ~12 rpm at intensities ≤ 50% of MDF or ~8 rpm at intensities > 50% of MDF would represent a performance change of ~5%.

This research is not exempt from limitations. Firstly, only trained male cyclists were included. Although it is hypothesized that the fit of the % of the MDF–cadence relationship would also be very strong in recreational and female cyclists, this aspect should be verified. Secondly, only one training stimulus (squat exercise) was used to examine the long-term stability of the % of the MDF–cadence relationship, so this stability should be examined after applying other stimuli like the so-called “torque” training. Finally, future studies should examine the mechanisms that could be behind the reduction in the linearity of the % of the MDF–cadence relationship at high intensities. Among others, changes in aspects like muscle recruitment and/or pedaling technique when pedaling at the maximal voluntariness against high resistances could explain this fact.

## 5. Practical Applications

We propose a relatively simple and economical procedure for the assessment of the force–velocity profile during cycling, as well as for the determination of a novel parameter such as the MDF. Our results showed that the % of the MDF–cadence relationship is reliable and stable over time, regardless of the changes in or different levels of cyclists’ MDF. In practice, the very close adjustment we found for this relationship would allow cyclists: (i) to determine the percentage of the MDF that is being used during every training or competition effort and (ii) to program the target cadence to train at a planned percentage of the MDF. The accuracy of these first two practical applications could even be maximized by using each cyclist’s individual relationship, thus reducing the slight between-subject differences associated with each % of the MDF. Moreover, the cadence achieved against the same load (in kp) pre-and post-training could be measured (iii) for practically quantifying changes in cyclist’s performance—e.g., the pre-post training differences of ~12 rpm at intensities < 50% of MDF or ~8 rpm at intensities > 50% of MDF would represent a performance change of ~5%.

## Figures and Tables

**Table 1:** Comparison of maximum torque values, average pedal cadence, and cadence associated with the maximum dynamic force (MDF) between subgroups of different MDF levels.

Subgroup	VO_2max_ (ml·kg^−1^·min^−1^)	MAP (W·kg^−1^)	MDF (N·kg^−1^)	Max Torque (N·m·kg^−1^)	Average Cadence (rpm) †	Cadence at MDF (rpm)
G1 (*n* = 17)	61.5 ± 1.6	5.2 ± 0.2	12.0 ± 0.8 ^#^	2.1 ± 0.1 ^#^	109 ± 7	22 ± 5
G2 (*n* = 17)	63.0 ± 1.9	5.4 ± 0.2	13.4 ± 0.4 *	2.3 ± 0.1 *	112 ± 10	22 ± 5
G3 (*n* = 18)	67.9 ± 2.9	5.9 ± 0.3	14.8 ± 0.6 *	2.6 ± 0.1 *^#^	111 ± 5	21 ± 3

G1, G2 and G3 are the groups based on MDF-tertiles (from lowest to highest). Abbreviations: MAP: maximal aerobic power, rpm: revolutions per minute. Symbols. * Significantly different from G1 (*p* < 0.05); ^#^ significantly different from G2 (*p* < 0.05); † average of all the force–cadence spectrum (from 10 to 100% MDF).

**Table 2:** Force–velocity profile (pedal cadence attained with each relative resistive force (% of maximal dynamic force [MDF], *n* = 52 participants) as follows: test–retest reliability (*n* = 10) and long-term stability after 10 weeks of combined aerobic and resistance (leg squat) training (*n* = 11).

Relative Resistive Force (% MDF)	Cadence (rpm)											
	General %MDF—Cadence Relationship		Test–Retest Reliability					Long-Term Stability				
			Day 1	Day 2	Difference (rpm)	SEM (rpm)	SEM (%)	Pre-Training	Post-Training	Difference (rpm)	SEM (rpm)	SEM (%)
	Mean ± SD	95% CI	Mean ± SD	Mean ± SD				Mean ± SD	Mean ± SD			
10	223 ± 15	219–227	222 ± 28	222 ± 23	0.9	7.5	3.4	223 ± 16	226 ± 11	−3.2	9.4	4.2
20	192 ± 12	189–196	192 ± 24	190 ± 21	1.1	5.3	2.8	194 ± 11	195 ± 7	−0.9	6.4	3.3
30	164 ± 11	161–167	163 ± 20	161 ± 19	1.4	4.4	2.7	167 ± 9	166 ± 7	0.6	4.3	2.6
40	138 ± 10	135–141	136 ± 17	135 ± 17	1.5	4.3	3.2	141 ± 10	139 ± 9	1.7	3.6	2.6
50	114 ± 10	111–117	112 ± 15	111 ± 14	1.0	4.6	4.1	117 ± 11	116 ± 10	1.8	3.6	3.1
60	92 ± 10	89–95	90 ± 13	89 ± 12	0.8	4.7	5.3	95 ± 11	93 ± 11	2.5	4.0	4.2
70	71 ± 9	69–74	70 ± 10	69 ± 9	0.8	4.5	6.5	74 ± 10	72 ± 10	2.8	4.0	5.5
80	53 ± 7	51–55	52 ± 8	51 ± 7	1.5	4.0	7.7	55 ± 9	53 ± 9	2.7	3.8	7.0
90	36 ± 5	35–38	36 ± 5	35 ± 4	1.2	3.2	9.0	38 ± 6	36 ± 6	2.2	3.2	8.6
100	22 ± 4	21–23	23 ± 4	22 ± 2	1.1	3.3	14.5	22 ± 6	21 ± 4	1.3	2.9	13.2

Abbreviations: CI, confidence interval; rpm, revolutions per minute; SD, standard deviation; SEM, standard error of the measurement.

## Data Availability

Data that support the findings of this study are available from the corresponding author upon reasonable request. The data are not publicly available due to privacy.
